# Supplementary material for: Causal interplay between lactose intolerance and gut microbiota: a combined bidirectional Mendelian randomization and in vivo validation study
Source: Front Nutr. 2026 Jun 1;13:1803337. doi: 10.3389/fnut.2026.1803337 (PMC13265576; doi:10.3389/fnut.2026.1803337)
Supplement: Supplementary file 1 [file Data_Sheet_1.zip › supplementary materials/Forward/leave one out cross validation/ebi-a-GCST90027527.finngen_R12_E4_LACTONAS.pdf]

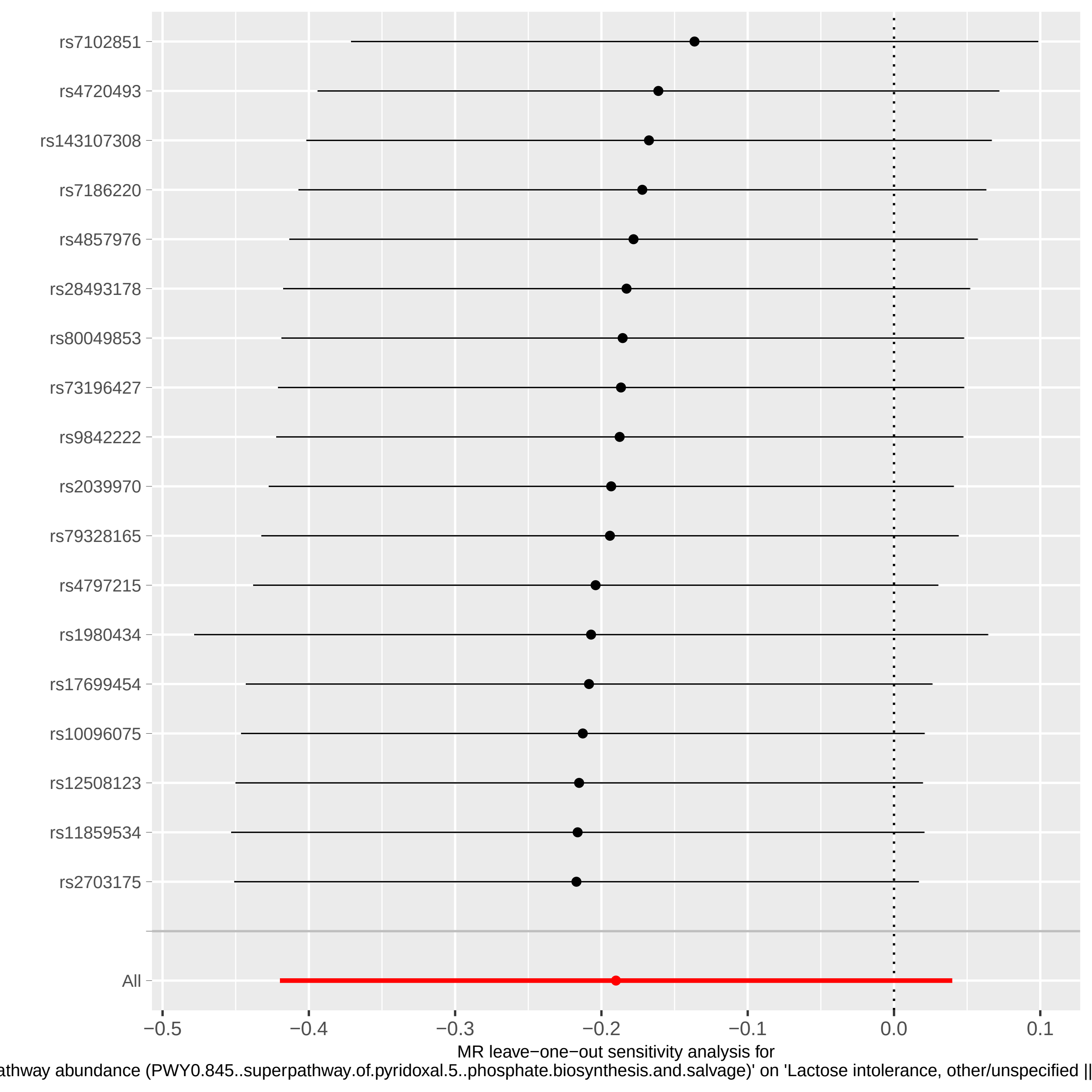
rs7102851
rs4720493
rs143107308
rs7186220
rs4857976
rs28493178
rs80049853
rs73196427
rs9842222
rs2039970
rs79328165
rs4797215
rs1980434
rs17699454
rs10096075
rs12508123
rs11859534
rs2703175
All
−0.5
−0.4
−0.3
−0.2
−0.1
0.0
0.1
MR leave−one−out sensitivity analysis for
athway abundance (PWY0.845..superpathway.of.pyridoxal.5..phosphate.biosynthesis.and.salvage)' on 'Lactose intolerance, other/unspecified ||
